# Validação de um Algoritmo Simples para Detecção de Taquicardia Ventricular no Eletrocardiograma

**DOI:** 10.36660/abc.20190501

**Published:** 2021-02-04

**Authors:** Francisco Santos, Cristiano F. Pisani, Francisco Carlos da Costa Darrieux, Celia M. F. Cirino, Denise Tessariol Hachul, Astrid M. Santos, Andrés Ricardo Pérez-Riera, Raimundo Barbosa-Barros, Mauricio Scanavacca

**Affiliations:** 1 Messejana - Dr. Carlos Alberto Studart Gomes (HM) Hospital FortalezaCE Brasil Messejana - Dr. Carlos Alberto Studart Gomes (HM) Hospital , Fortaleza , CE - Brasil; 2 Universidade de São Paulo Faculdade de Medicina Hospital das Clínicas São PauloSP Brasil Universidade de São Paulo Faculdade de Medicina Hospital das Clínicas Instituto do Coração, São Paulo , SP - Brasil; 3 Faculdade de Medicina do ABC São PauloSP Brasil Faculdade de Medicina do ABC , São Paulo , SP – Brasil

**Keywords:** Taquicardia Supraventricular, Taquicardia Ventricular, Arritmias Cardíacas, Eletrocardiografia

## Abstract

**Fundamento:**

O diagnóstico diferencial de taquicardia de QRS largo, entre taquicardia ventricular (TV) ou taquicardia supraventricular com condução aberrante (TSV-A) é algumas vezes difícil de ser feito na sala de emergência.

**Objetivo:**

Avaliar a acurácia de um algoritmo novo e simples para a detecção de TV no eletrocardiograma (ECG) em pacientes com taquicardia de QRS largo.

**Métodos:**

ECGs de 12 derivações para detecção de taquicardia de QRS largo foram obtidos prospectivamente de 120 pacientes durante estudo eletrofisiológico. Seis médicos com diferentes experiências analisaram os ECGs, e fizeram o diagnóstico com base no algoritmo D12V16, que envolve a análise da polaridade predominante do complexo QRS nas derivações I, II, V1 e V6. O diagnóstico foi comparado com os obtidos pelo algoritmo tradicional de Brugada e pelo estudo eletrofisiológico, o qual é considerado padrão ouro. Adotou-se um nível de significância de 5% (p<0,05) nas análises estatísticas.

**Resultados:**

De acordo com o estudo eletrofisiológico, 82 ECGs eram de TV e 38 de TSV-A. Doenças cardíacas estruturais estavam presentes em 71 (86,6%) dos pacientes com TV e em oito (21,1%) com TSV-A. O algoritmo de Brugada teve uma maior sensibilidade global (87,2%), enquanto o algoritmo D12V16 apresentou maior especificidade global (85,1%) para TV. Tanto o algoritmo D12V16 como o de Brugada apresentou um alto valor preditivo positivo (90,9% vs. 85,8%, respectivamente) e acurácia similar (73,8% vs. 81,4%, respectivamente) para o diagnóstico de TV. Nos avaliadores experientes, a acurácia foi maior utilizando o algoritmo de Brugada que o algoritmo D12V16, mas a acurácia dos dois algoritmos foi similar segundo os avaliadores menos experientes.

**Conclusão:**

O algoritmo simplificado pode ser um método útil para reconhecer TV no ECG, principalmente para médicos menos experientes. (Arq Bras Cardiol. 2021; [online].ahead print, PP.0-0)

## Introdução

O reconhecimento da taquicardia ventricular (TV) como mecanismo de taquicardia de QRS largo é uma importante questão, uma vez que uma análise incorreta do eletrocardiograma (ECG) pode levar à terapia inadequada. O diagnóstico de TV também permite o manejo agudo e de longo prazo mais apropriado do paciente e previne internações hospitalares e exames desnecessários. ^1 , 2^

Desde os anos 60, muitos critérios eletrocardiográficos foram propostos na tentativa de se diferenciar TV de taquicardia supraventricular com condução aberrante (TSV-A). ^3 - 10^ Muitos deles consideram que medidas específicas em milissegundos resultam em dificuldade de memorização, baixa reprodutibilidade da acurácia, e baixa aplicabilidade clínica. Ainda, na medicina de emergência, há uma alta taxa de discordância entre observadores, com uma baixa acurácia diagnóstica, ^11 - 13^ que poderia levar à uma terapia prejudicial. ^14^

Assim, o desenvolvimento de um método simples, preferivelmente visual e de fácil memorização por médicos residentes do departamento de emergência, independentemente de experiência em arritmia, poderia melhorar as decisões clínicas.

O poder discriminatório das derivações I, II, V1 e V6 para identificar os mecanismos da taquicardia de QRS largo foi inicialmente proposto por Nagi et al., ^15^ mas não foi validado prospectivamente. O objetivo deste estudo foi avaliar a acurácia diagnóstica de um método simples, de fácil memorização, para o diagnóstico diferencial da taquicardia de QRS largo.

## Métodos

O protocolo de pesquisa foi aprovado pelo Comitê Científico do do Instituto do Coração (InCor) do HC-FMUSP, e pelo comitê de ética em pesquisa do Messejana - Dr. Carlos Alberto Studart Gomes (HM) - Hospital sob o protocolo número 336.107.

### Pacientes e ECGs

Nós selecionamos eletrocardiogramas de 120 pacientes consecutivos (um de cada paciente) que apresentaram taquicardia de QRS largo no estudo eletrofisiológico (EEF) realizado em nosso centro entre janeiro de 2007 e dezembro de 2013. O padrão ouro para definir o mecanismo da taquicardia foi o estudo eletrofisiológico (EEF).

Após receberem informações sobre o estudo, os pacientes que aceitaram a participar do estudo assinaram um termo de consentimento. O estudo foi conduzido de acordo com os princípios éticos em pesquisa envolvendo humanos.

A taquicardia de QRS largo foi definida por EEF, como uma frequência cardíaca ≥ 100 bpm, na ausência de onda P sinusal conduzida visível e duração do complexo QRS ≥ 120ms. Esses traçados foram registrados no EEF e obtidos de maneira prospectiva e consecutiva. Pacientes com taquicardia de QRS largo em uso de medicamentos antiarrítmicos, pacientes com taquicardia pré-excitada, e pacientes com marca-passos artificiais foram excluídos. Após seleção dos ECGs de 12 derivações, nós coletamos as seguintes variáveis clínicas de cada paciente: idade, sexo, e presença ou ausência de doença cardíaca estrutural.

### Algoritmo D12V16 baseado na análise das derivações I, II, V1 e V6

**Figura 1:**
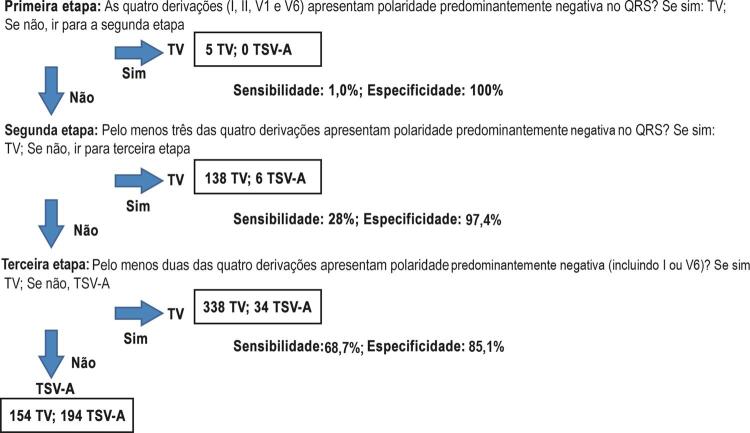
– Algoritmo simplificado das derivações I, II, V1 e V6. Análise de 120 eletrocardiograma para taquicardia com QRS largo realizada pelos seis avaliadores (total de 720 análises). Cada avaliador analisou 82 taquicardias ventriculares e 38 taquicardias ventriculares com condução aberrante. Para cada etapa, são apresentados sensibilidade, especificidade e número de eletrocardiogramas que preencheram o diagnóstico; TV: taquicardia ventricular; TSV-A: taquicardia supraventricular de condução aberrante.

**Figura 2:**
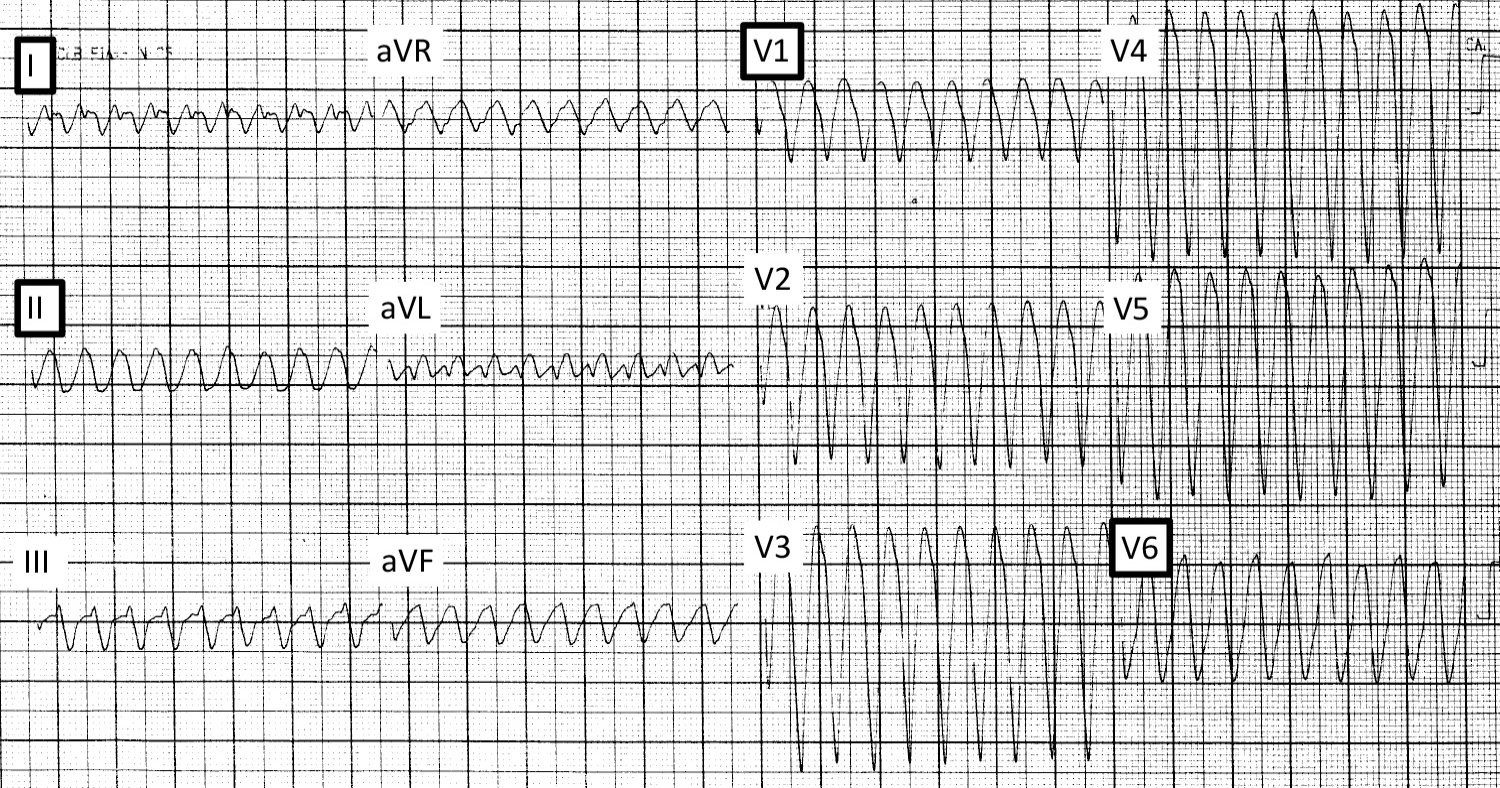
– Exemplos das quatro derivações com polaridade predominantemente negativa (Etapa 1). Esses achados têm 100% de especificidade para o diagnóstico de taquicardia ventricular.

**Figura 3:**
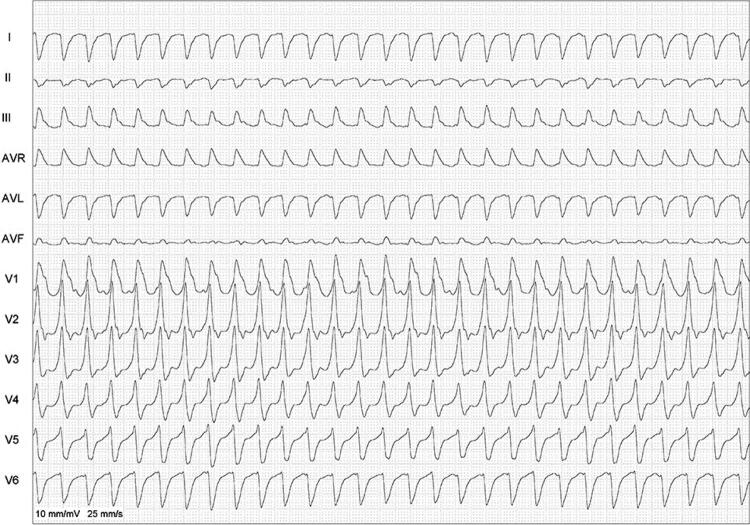
– Taquicardia de QRS largo, usando o algoritmo simplificado; observa-se polaridade predominantemente negativa de três das quatro derivações, sugerindo taquicardia ventricular.

Todos os ECGs foram analisados de acordo com o algoritmo simplificado, em três etapas ( Figura 1 ): (1) na primeira etapa, TV foi considerada se as quatro derivações (I, II, V1 e V6) apresentassem uma polaridade predominantemente negativa (razão R/S <1). Na ausência desses achados, nós prosseguimos para a etapa seguinte. (2) Na segunda etapa, a TV foi diagnosticada se pelo menos três das quatro derivações apresentassem polaridade predominantemente negativa. Se essa etapa não fosse completada, nós prosseguimos para a etapa seguinte. (3) Na terceira etapa, o diagnóstico de TV foi definido se pelo menos duas das quatro derivações apresentassem polaridade predominantemente negativa (I ou V6 necessariamente incluídas). Se essas etapas não fossem totalmente completadas, assumia-se o diagnóstico de TSV-A. As Figuras 2 , 3 e 4 apresentam todas as etapas do algoritmo.

**Figura 4:**
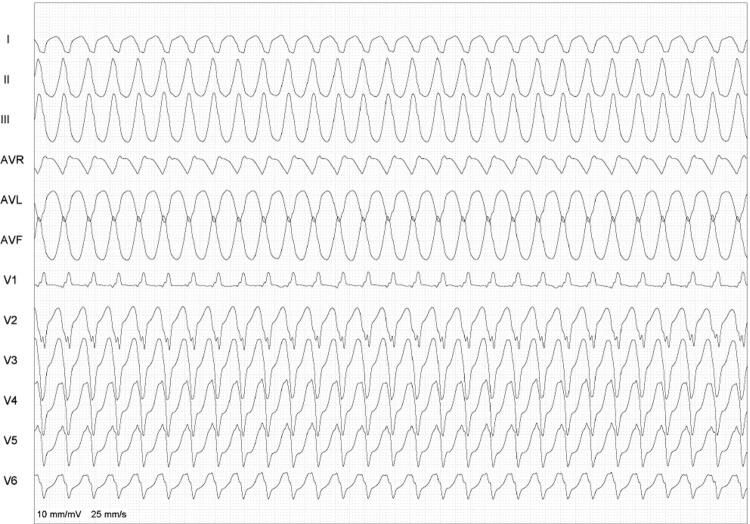
– Exemplo de duas polaridades predominantemente negativas (etapa 3) em quatro derivações (DI, DII, V1 e V6), com inclusão de DI e/ou V6 (neste caso, ambas negativas).

### Avaliadores e análise dos ECGs

Os ECGs foram analisados por três pares de investigadores: (1) dois cardiologistas com expertise em arritmia clínica; (2) dois cardiologistas clínicos; e (3) dois médicos residentes do departamento de emergência. O uso de lente de aumento foi permitido se necessário.

Os ECGs foram analisados pelos seis examinadores em quatro estágios diferentes: (A) usando o algoritmo de Brugada; ^6^ (B) usando o algoritmo D12V16; (C) repetindo-se a análise usando o algoritmo de Brugada com informação clínica; e (D) repetindo-se a análise usando o algoritmo D12V16 com informação clínica.

Todos os ECGs foram distribuídos aleatoriamente, e todos os examinadores eram cegos quanto ao diagnóstico feito pelo EEF. Informações clínicas de cada ECG foram reveladas aos examinadores após completarem os estágios A e B.

### Análise estatística

As características dos pacientes estão descritas de acordo com o diagnóstico por EEF usando frequências absolutas e relativas. A associação entre cada característica e o diagnóstico foi confirmada pelo teste do qui-quadrado. A idade do paciente foi descrita por média e desvio padrão e comparada segundo o diagnóstico pelo teste *t* não pareado de Student.

Os diagnósticos foram descritos de acordo com as quatro etapas de análise do ECG, e os resultados obtidos foram comparados com o EEF. Os seguintes parâmetros diagnósticos foram calculados – sensibilidade (S), especificidade (Es), valor preditivo positivo (VPP), valor preditivo negativo (VPN) e acurácia. A concordância de cada etapa com o EEF foi avaliada pelo coeficiente kappa. Calculamos, ainda, o intervalo de confiança de 95% (IC95%) para todas as medidas estimadas.

As análises foram repetidas somente para os diagnósticos finais, e os grupos separados de acordo com a experiência do examinador. Para análise estatística de S e Es, usamos o total de ECGs (n=120) interpretados pelos seis examinadores. Concordância e discordância com o EEF, considerado o método padrão-ouro, foram estabelecidas para cada procedimento, enquanto a associação marginal entre os métodos foi avaliada pelo teste de McNemar. Concordância e discordância entre avaliadores foram determinadas em ambos os métodos e esses procedimentos comparados pelo teste de McNemar Comparamos ambos os métodos entre os grupos de avaliadores (variabilidade do método tradicional entre diferentes avaliadores). O teste Kolmogorov-Smirnov confirmou a normalidade da distribuição da idade. O nível de significância foi estabelecido em 5% (p<0,05). As análises foram realizadas usando o programa IBM-SPSS para Windows, versão 20.0.

## Resultados

### Características dos pacientes

As características dos pacientes estão apresentadas na Tabela 1 . Segundo o EEF, 82 pacientes apresentaram TV (68%), e 38 apresentaram TSV-A; 81 pacientes eram do sexo masculino, e 69,5% desses apresentaram TV e 63,2% TSV-A. A idade média dos pacientes foi 49,1 ± 17,5 anos. Os pacientes com TV eram mais velhos que os pacientes com TSV-A (52,7±16,3 vs. 41,4±17,8 anos; p= 0,001). Doença cardíaca estrutural foi detectada em 79 (65,8%) dos 120 pacientes, em 71(86,6%) dos 82 pacientes com TV, e oito (21,1%) dos 38 pacientes com TSV-A. As características dos pacientes estão apresentadas na Tabela 1 .

**Tabela 1:** Características clínicas dos pacientes diagnosticados com taquicardia supraventricular com aberrância de condução (TSV-A) e taquicardia ventricular (TV) de acordo com estudo eletrofisiológico

Variável	TSV-A (n=38)	TV (n=82)	Total (n=120)	P
Sexo masculino, n (%)	24 (63,2)	57 (69,5)	81 (67,5)	0,489
Doença cardíaca, n (%)	8 (21,1)	71 (86,6)	79 (65,8)	<0,001
Idade, anos	41,4±17,8	52,7±16,3	49,1±17,5	0,001*

Teste do qui-quadrado; *teste t de Student.

### Análise global dos algoritmos

Valores de S, Es, VPP, VPN e de acurácia, de acordo com cada etapa do algoritmo, são apresentados na Tabela 2 . Os seis examinadores realizaram um total de 2880 análises (720 usando o algoritmo simplificado e o algoritmo de Brugada, independentemente das informações clínicas). A S do algoritmo de Brugada foi maior que a do algoritmo simplificado (87,2% vs. 68,7%), ao passo que a Es do algoritmo D12V16 foi maior que a do algoritmo de Brugada (85,1% vs. 68,9%). O VPP para TV foi alto para ambos os métodos (90,9% no algoritmo D12V16 e 85,8% no algoritmo de Brugada). A acurácia diagnóstica foi de 73,8% usando o algoritmo D12V16 (k = 0,471; IC 95% 0,41–0,53), e de 81,4% usando o algoritmo de Brugada (k = 0,566; IC95%, 0,5–0,63). Apesar de não significativo, observou-se um aumento na Es na presença de informação clínica (90,8% para o algoritmo simplificado, 73,7% para o algoritmo de Brugada). Houve um pequeno aumento na acurácia quando o algoritmo de Brugada foi aplicado na presença de informação clínica (k = 0,607; IC 95%, 0,54–0,67). Não se observou aumento na acurácia do algoritmo D12V16 na presença ou ausência de informação clínica (k = 0,471; IC95%, 0,41–0,53; e k = 0,474; IC95%, 0,42–0,53, respectivamente).

**Tabela 2:** – Sensibilidade, especificidade, valor preditivo positivo (VPP), valor preditivo negativo (VPN), e acurácia de cada etapa do algoritmo de Brugada e do algoritmo D12V16, com e sem características clínicas

Critério	Etapa	Sensibilidade para diagnóstico de TV, % (IC95%)	Especificidade para diagnóstico de TV, % (IC95%)	VPP para diagnóstico de TV, % (IC95%)	VNP para diagnóstico de TV, % (IC95%)	Acurácia, % (IC95%)
Algoritmo de Brugada	Etapa 1	19,7 (16,3-23,5)	91,7 (87,3-94,9)	83,6 (75,6-89,8)	34,6 (30,8-38,5)	42,5 (38,9-46,1)
	Etapa 2	48,0 (43,5-52,5)	86,8 (81,8-90,9)	88,7 (84,3-92,3)	43,6 (39,0-48,3)	60,3 (56,7-63,9)
	Etapa 3	61,8 (57,3-66,1)	83,8 (78,3-88,3)	89,1 (85,4-92,2)	50,4 (45,2-55,5)	68,7 (65,3-72,1)
	Etapa 4	87,2 (83,9-90,0)	68,9 (62,4-74,8)	85,8 (82,4-88,7)	71,4 (64,9-77,2)	81,4 (78,6-84,2)
Algoritmo D12V16	Etapa 1	1,0 (0,3-2,4)	100 (98,4-99,0)	100 (47,8-100,0)	31,9 (28,5-42,7)	32,4 (29,0-35,8)
	Etapa 2	28,0 (24,1-32,2)	97,4 (94,4-99,0)	95,8 (91,2-98,5)	38,5 (34,5-42,7)	50,0 (46,3-53,7)
	Etapa 3	68,7 (64,4-72,8)	85,1 (79,8-89,4)	90,9 (87,5-93,6)	55,7 (50,4-61,0)	73,8 (70,6-77,0)
Brugada com informações clínicas	Etapa 1	28,0 (24,1-32,2)	89 (84,2-92,8)	84,7 (78,2-89,8)	36,4 (32,4-40,6)	47,4 (43,8-51,0)
	Etapa 2	49,4 (44,9-53,9)	87,3 (82,2-91,3)	89,3 (85,0-92,7)	44,4 (39,8-49,2)	61,4 (57,8-65,0)
	Etapa 3	62,0 (57,5-66,3)	83,8 (78,3-88,3)	89,2 (85,4-92,3)	50,5 (45,4-55,7)	68,9 (65,5-72,3)
	Etapa 4	87,2 (83,9-90,0)	73,7 (67,5-79,3)	87,7 (84,5-90,5)	72,7 (66,5-78,4)	82,9 (80,1-85,7)
D12V16 com informações clínicas	Etapa 1	1,4 (0,6-2,9)	100 (98,4-100,0)	100 (59,0-100,0)	32,0 (28,6-35,5)	32,7 (29,3-36,1)
	Etapa 2	27,4 (23,5-31,6)	98,7 (96,2-99,7)	97,8 (93,8-99,5)	38,7 (34,7-42,8)	50,0 (46,3-53,7)
	Etapa 3	65,0 (60,6-69,3)	90,8 (86,3-94,2)	93,8 (90,7-96,1)	54,6 (49,5-59,7)	73,1 (69,9-76,3)

*Valores expressos em % (intervalo de confiança de 95%), TV: taquicardia ventricular.*

### Resultados das análises por grupos de examinadores

Valores de S, Es, VPP, VPN e de acurácia, de acordo com cada grupo de avaliadores estão apresentados na Tabela 3 . Em todos os três grupos, observou-se uma maior S para o algoritmo de Brugada em comparação ao algoritmo D12V16, sem diferença entre os grupos. No entanto, a Es foi bem mais evidente no grupo de médicos residentes do departamento de emergência (grupo 3) em comparação aos outros grupos. Uma maior acurácia diagnóstica foi obtida quando avaliadores especialistas em cardiologia (grupos 1 e 2) usaram o algoritmo de Brugada comparativamente ao algoritmo D12V16 (84,6% e 85.8% vs. 74,2% e 74,6%, respectivamente). Para o grupo 3, a acurácia diagnóstica dos dois métodos foi similar (73,7% usando o algoritmo de Brugada, 72,9% usando o algoritmo simplificado). Para esses avaliadores, o algoritmo D12V16 mostrou maior Es que o algoritmo de Brugada (85,5% e 65,8%, respectivamente). Esses valores não foram significativamente diferentes na presença de informação clínica ( Tabela 4 ).

**Tabela 3:** – Sensibilidade, especificidade, valor preditivo positivo (VPP), valor preditivo negativo (VPN), e acurácia de cada grupo de avaliadores

Critério	Grupo	Sensibilidade para diagnóstico de TV, % (IC95%)	Especificidade para diagnóstico de TV, % (IC95%)	VPP para diagnóstico de TV, % (IC95%)	VNP para diagnóstico de TV, % (IC95%)	Accuracy, % (95% CI)
Brugada	I – Expertise em arritmias	88,4 (82,5-92,9)	76,3 (65,2-85,3)	89,0 (83,1-93,3)	75,3 (64,2-84,4)	84,6 (82,0-87,2)
	II – Cardiologistas clínicos	95,7 (91,4-98,3)	64,5 (52,7-75,1)	85,3 (79,4-90,1)	87,5 (75,9-94,8)	85,8 (83,3-88,3)
	III- Médicos residentes de Emergência	77,4(70,3-83,6)	65,8 (54,0-76,3)	83,0 (76,1-88,6)	57,5 (46,4-68,0)	73,7 (70,5-76,9)
D12V16 Algorithm	I – Expertise em arritmias	68,9 (61,2-75,9)	85,5 (75,6-92,5)	91,1 (84,7-95,5)	56,0 (46,5-65,2)	74,2 (71,0-77,4)
	II – Cardiologistas clínicos	70,1 (62,5-77,0)	84,2 (74,0-91,6)	90,6 (84,1-95,0)	56,6 (47,0-65,9)	74,6 (71,4-77,8)
	III- Médicos residentes de Emergência	67,1 (59,3-74,2)	85,5 (75,6-92,5)	90,9 (84,3-95,4)	54,6 (45,2-63,8)	72,9 (69,7-76,1)
Brugada com informação clínica	I – Expertise em arritmias	86 (79,7-90,9)	81,6 (71,0-89,5)	91,0 (85,3-95,0)	72,9 (62,2-82,0)	84,6 (82,0-87,2)
	II – Cardiologistas clínicos	98,2 (94,7-99,6)	72,4 (60,9-82,0)	88,5 (82,9-92,7)	94,8 (85,6-98,9)	90,0 (87,8-92,2)
	III- Médicos residentes de Emergência	77,4 (70,3-83,6)	67,1 (55,4-77,5)	83,6 (76,7-89,1)	58,0 (47,0-68,4)	74,1 (70,9-77,3)
D12V16 com Informação clínica	I – Expertise em arritmias	65,2 (57,4-72,5)	94,7 (87,1-98,5)	96,4 (91,0-99,0)	55,8 (46,8-64,5)	74,6 (71,4-77,8)
	II – Cardiologistas clínicos	70,1 (62,5-77,0)	88,2 (78,7-94,4)	92,7 (86,7-96,6)	57,8 (48,2-66,9)	75,8 (72,7-78,9)
	III- Médicos residentes de Emergência	59,8 (51,8-67,3)	89,5 (80,3-95,3)	92,5 (85,7-96,7)	50,7 (42,0-59,5)	69,1 (65,7-72,5)

*Valores expressos em % (intervalo de confiança de 95%).*

**Tabela 4:** – Concordância e discordância entre os diagnósticos feitos com o algoritmo de Brugada e o algoritmo D12V16

Avaliação	Brugada	D12V16	p
**Sem informação clínica**			<0,001
Concordância para TSV-A, n (%)	157 (21,8)	194 (26,9)	
Concordância para TV, n (%)	429 (59,6)	338 (46,9)	
Discordância, n (%)	134 (18,6)	188 (26,2)	
**Com informação clínica**			<0,001
Concordância para TSV-A, n (%)	168 (23,3)	207 (28,7)	
Concordância para TV, n (%)	429 (59,6)	320 (44,4)	
Discordância, n (%)	123 (17,1)	193 (26,9)	

*Resultados obtidos pelo teste de McNemar; TV: taquicardia ventricular; TSV-A: taquicardia supraventricular com condução aberrante.*

### Concordância diagnóstica entre avaliadores e métodos

**Figura 5:**
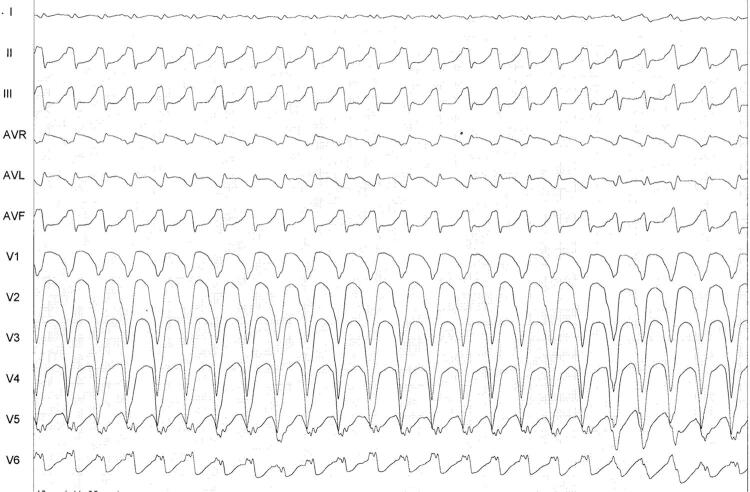
– Exemplo de taquicardia de QRS largo com discordância quanto ao diagnóstico – enquanto o algoritmo simplificado sugere taquicardia supraventricular com aberração de condução (TSV-A) (etapa 3), o algoritmo de Brugada sugere taquicardia ventricular (etapa 4).

Dados sobre concordância diagnóstica entre os seis avaliadores, baseada na análise dos 120 ECGs são apresentados na Tabela 5 . A porcentagem de discordância usando o algoritmo de Brugada e o algoritmo D12V16 foi de 60,8% e 30%, respectivamente. Na presença de informação clínica, esses valores foram 51,7% e 30,8%, respectivamente. Essa diferença foi estatisticamente significativa (p<0,001), com ou sem informação clínica. A Figura 5 mostra a discordância entre os algoritmos.

**Tabela 5:** – Concordância e discordância nos diagnósticos realizados por seis avaliadores que analisaram 120 eletrocardiogramas usando o algoritmo de Brugada e o algoritmo D12V16

Avaliação	Brugada	D12V16	p
**Sem informação clínica**			<0,001
Concordância para TSV-A, n (%)	8 (6,7)	40 (33,3)	
Concordância para TV, n (%)	39 (32,5)	44 (36,7)	
Discordância, n (%)	73 (60,8)	36 (30,0)	
**Com informação clínica**			<0,001
Concordância para TSV-A, n (%)	18 (15,0)	43 (35,8)	
Concordância para TV, n (%)	40 (33,3)	40 (33,4)	
Discordância, n (%)	62 (51,7)	37 (30,8)	

*Resultados obtidos pelo teste de McNemar; TV: taquicardia ventricular; TSV-A: taquicardia supraventricular com condução aberrante.*

## Discussão

Muitos dos critérios usados para o diagnóstico diferencial de taquicardia de QRS largo são baseados em aspectos peculiares do ECG, os quais são de difícil memorização, comprometendo a aplicabilidade clínica e consequentemente levando a uma falta de reprodutibilidade. ^7 , 11^ Sempre que possível, recomenda-se a consulta a especialistas para reduzir erros diagnósticos e consequências adversas. ^2^ Contudo, não se pode contar com a presença de especialistas em todos os departamentos de emergência.

Em 1999, Nagi et al., ^15^ propuseram valores de derivações bipolares (I-II) e de derivações precordiais (V1-V6) para o diagnóstico diferencial de taquicardia de QRS largo, guiado por um software, para análise do ECG. A predominância de polaridade negativa de pelo menos duas das quatro derivações, com inclusão da derivação I ou V6, fez o diagnóstico de VT em 89,2% dos casos. Apesar de ser uma técnica facilmente aplicada por não especialistas, o método não foi avaliado sistematicamente. Em nosso estudo, comparamos o diagnóstico obtido por análise da polaridade das derivações DI, DII, V1 e V6 (algoritmo D12V16) com o algoritmo mais usado para o diagnóstico de taquicardia de QRS (algoritmo de Brugada), em um número significativo de pacientes. Também avaliamos o papel da informação clínica para melhorar a taxa de diagnóstico correto, aplicado pelos médicos, independentemente de sua expertise em arritmia. A Es do método D12V16 foi maior para o diagnóstico de TV e houve coeficientes de discordância menores (maior reprodutibilidade) entre observadores, com acurácia similar à do algoritmo de Brugada. Deve-se enfatizar que a inclusão das etapas 1 e 2, diferentemente do estudo original de Nagi et al., ^15^ aumentou a Es do algoritmo simplificado (100% e 97%, respectivamente), fornecendo, assim, resultados comparáveis com a presença de dissociação atrioventricular, ^16 , 17^ e com as etapas 1 e 2 do algoritmo de Brugada, como relatado no trabalho original. ^6^

Não é de se surpreender que não foi possível reproduzir a elevada acurácia, S, e Es descrita por Brugada et al., ^6^ como previamente relatado por outros autores. ^4 , 7 , 11 , 18^ Uma das razões pode ser o fato de que os avaliadores que desenvolveram o algoritmo de Brugada eram bem mais experientes que os de outros estudos. Esses achados foram confirmados em nosso estudo, em que a acurácia diagnóstica obtida por cardiologistas usando o algoritmo de Brugada foi maior que aquela obtida por médicos menos experientes em arritmia ou cardiologia (84,6%; 85,8% e 73,7% para os grupos I, II, e III, respectivamente). No grupo III, os dois critérios apesentaram resultados similares em termos de acurácia diagnóstica (73,7% usando o algoritmo de Brugada e 72,9% usando o algoritmo simplificado). Além disso, os ECGs também em nosso estudo podem também ser diferentes daqueles apresentados no estudo de Brugada et al., ^6^ em que não foram obtidas informações sobre pacientes apresentado bloqueio de ramo prévio, TV idiopática, TSV pré-excitada, ou TSV-A, situações que não foram investigadas neste estudo. ^19 , 20^

Apesar de uma história de doença cardíaca estrutural (infarto do miocárdio, angina pectoris, insuficiência cardíaca congestiva) ter resultado em um alto VPP para TV, ^21^ não houve aumento significativo na acurácia quando presença de informação clínica foi adicionada à análise usando ambos os métodos eletrocardiográficos. Ao se comparar com o algoritmo de Brugada segundo presença ou ausência de informação clínica (k = 0,566; IC95%, 0,5–0,63 e k = 0,607, IC95%, 0,54–0,67, respectivamente), nós observamos um pequeno aumento no diagnóstico. Uma possível explicação é o fato de que, quando a informação clínica foi adicionada aos ECGs considerados “limítrofes” (“ *borderline* ”) pela análise de Brugada, os avaliadores tiveram um reforço para o diagnóstico final. Esse fato não foi observado para o algoritmo simplificado, possivelmente porque a análise da interpretação proposta dos parâmetros eletrocardiográficos teve um apelo visual, que não demandou uma análise extensa.

A TV é predominante em pacientes com taquicardia de QRS largo. ^8 , 22^ Em nosso estudo, a prevalência de TV foi de 68%, enquanto doença cardíaca estrutural foi detectada em 87% dos pacientes, confirmando os resultados de outros estudos. ^5 , 21 , 23^ Apesar de o algoritmo D12V16 e o algoritmo de Brugada terem apresentado acurácia similar para o diagnóstico de TV (taxas de 73,8% e 81,4% respectivamente), eles divergiram em termos de S e Es. Assim, diferentes critérios diagnósticos para TV ou TSV-A têm diferentes valores diagnósticos. Por exemplo, um avaliador provavelmente não errará no diagnóstico de TV usando o algoritmo de Brugada, dada a sua alta S (87,2%). Por outro lado, é improvável que a TV seria diagnosticada erroneamente usando o algoritmo D12V16 dada sua alta Es (85,1%).

A maior discrepância esteve relacionada com a Es do método de Brugada (68,9% em nosso estudo vs. 96,5% no estudo original publicado pelos autores). ^6^ Lau et al., ^22^ Vereckei et al., ^8^ e Griffith et al., ^4^ também apresentaram menores valores de Es usando o algoritmo de Brugada (44%, 73.3%, e 67%, respectivamente) em comparação ao estudo de Brugada et al. ^6^

A maior S do algoritmo de Brugada tem implicações clínicas importantes no manejo agudo, uma vez que diminui a possibilidade de que pacientes com TV sejam tratados como TSV-A, o que levaria a consequências deletérias. ^2^ Por outro lado, o maior VPP (95,8%) da segunda etapa do algoritmo D12V16 poderia ter implicações futuras na tomada de decisões na prática clínica e no desenvolvimento de outros algoritmos discriminatórios para melhorar o desempenho diagnóstico. No entanto, deve-se enfatizar que, quando os critérios do algoritmo D12V16 não são preenchidos para TV, não se pode concluir que a TSV-A seja o diagnóstico final, dada a relativa baixa S (68,7%) desse novo algoritmo.

Herbert et al., ^11^ investigaram a variabilidade entre observadores para diferenciar TV de TSV-A usando o algoritmo de Brugada na medicina de emergência. Uma discrepância de 22% foi observada na prevalência diagnóstica. Em nosso estudo, entre os seis avaliadores, detectou-se uma maior porcentagem de discordância com o uso do algoritmo de Brugada em comparação ao algoritmo D12V16 (60,8% e 30%, respectivamente). Essa diferença foi estatisticamente significativa (p<0,001), e não houve redução na presença de informação clínica (51,7%, e 30,8% respectivamente; p < 0,001). Esses resultados poderiam ser explicados pelo fato de o algoritmo D12V16 ser uma metodologia mais simples, de três etapas.

Em resumo, este estudo avaliou um algoritmo simples para o diagnóstico de taquicardia de QRS largo e o comparou com o tradicional algoritmo de Brugada. ^6^ Avaliadores com experiências distintas realizaram a análise com ou sem informação clínica. Tanto o algoritmo D12V16 como o algoritmo de Brugada apresentaram acurácia similar para o diagnóstico diferencial de taquicardia de QRS largo. O algoritmo de Brugada mostrou-se mais eficiente quando aplicado por cardiologistas (grupos I e II) em comparação ao algoritmo D12V16, diferença que não foi observada na análise dos médicos residentes da emergência (grupo III). As etapas 1 e 2 do algoritmo simplificado tiveram um alto VPP para diagnóstico de TV. A Es e a concordância entre examinadores foram maiores ao usar o algoritmo D12V16. Ainda, a acurácia diagnóstica dos dois métodos não aumentou significativamente com a disponibilidade da informação clínica (presença ou ausência de doença cardíaca).

### Limitações

Uma das principais limitações do estudo foi o fato de ter sido conduzido em um ambiente fora do contexto de emergência, e os avaliadores não tiveram restrição de tempo para fazer o diagnóstico, eliminando, assim, fatores como estresse e decisões rápidas, situações comuns na sala de emergência. Os avaliadores também analisaram todas as derivações dos ECGs e não somente derivações específicas, como as derivações bipolares I-II e precordiais V1-V6, quando usaram o algoritmo simplificado, ou as derivações V1-V6 quando usaram o algoritmo de Brugada. Apesar de esse ser o método usado no “mundo real”, essa visão global do ECG poderia influenciar o diagnóstico diferencial. Como a S final do algoritmo foi 68,7% para TV quando todos os três critérios para TV foram negativos, o erro no diagnóstico de TV como TSV-A poderia levar a um tratamento deletério no contexto de emergência se somente esse algoritmo fosse utilizado.

O número de avaliadores em cada grupo foi pequeno e cada um analisou cada ECG mais de uma vez. Nós não analisamos a variabilidade diagnóstica entre os observadores dos mesmos grupos e entre os diferentes momentos da análise do ECG. Tal fato poderia limitar a interpretação dos resultados.

Ainda, outras possíveis causas de taquicardia de complexo QRS largo foram excluídas, tais como a TSV antidrômica, TV mediada por marca-passo, hipercalemia ou outros distúrbios eletrolíticos, toxicidade a antiarrítmicos das classes IA e IC, e uso/abuso de antidepressivos tricíclicos. Também excluímos pacientes pediátricos com taquicardia de complexo QRS largo da análise. Não comparamos o algoritmo D12V16 com outros algoritmos, tais como o critério de Pava, ^24^ os dois algoritmos de Vereckei, ^8 , 9^ a abordagem Bayesiana, ^25^ e o mais recente escore de TV. ^10^ A validação externa desse algoritmo simplificado poderia trazer informação adicional sobre a consistência desse algoritmo.

## Conclusões

O algoritmo D12V16 é um algoritmo simples, de três etapas, que demonstrou alta Es e elevado VPP para o diagnóstico de TV e pode representar um instrumento simples e útil no reconhecimento de TV em pacientes com taquicardia de complexo QRS largo. Quando analisado por avaliadores menos experientes, o algoritmo mostrou acurácia similar em comparação ao algoritmo de Brugada. No entanto, deve-se enfatizar que, quando as etapas do algoritmo D12V16 não são preenchidas para TV, não se pode concluir que a TSV-A seja o diagnóstico final, dada sua baixa S (68,7%) e portanto, o risco de se “perder” alguns diagnósticos de TV. Outros estudos devem ser conduzidos para validar os resultados.
