# Supplementary material for: Validação de um Algoritmo Simples para Detecção de Taquicardia Ventricular no Eletrocardiograma
Source: Arq Bras Cardiol. 2021 Feb 4;116(3):454–63. [Article in Portuguese] doi: 10.36660/abc.20190501 (PMC8159554; doi:10.36660/abc.20190501)
Supplement: Translation

# Translation: Validation of a Simple Electrocardiographic Algorithm for Detection of Ventricular Tachycardia

**Authors:** Francisco Santos, Cristiano F. Pisani, Francisco Carlos da Costa Darrieux, Celia M. F. Cirino, Denise Tessariol Hachul, Astrid M. Santos, Andrés Ricardo Pérez-Riera, Raimundo Barbosa-Barros, Mauricio Scanavacca

**Affiliations:** 1 Brazil Messejana - Dr. Carlos Alberto Studart Gomes (HM) Hospital, Fortaleza, CE - Brazil; 2 Brazil Universidade de São Paulo Faculdade de Medicina Hospital das Clínicas Instituto do Coração, São Paulo, SP - Brazil; 3 Brazil Faculdade de Medicina do ABC, São Paulo, SP – Brazil

**Keywords:** Tachycardia Supraventricular, Tachycardia Ventricular, Arrhythmias Cardiac, Electrocardiography

## Abstract

**Background:**

The differential diagnosis of wide QRS complex tachycardia (WCT) between ventricular tachycardia (VT) or supraventricular tachycardia with aberrant conduction (SVT-A) is sometimes difficult in the emergency room.

**Objective:**

The aim of this study was to evaluate the accuracy of a new simple electrocardiographic algorithm to recognize VT in patients with wide complex tachycardia.

**Methods:**

The 12-lead electrocardiograms (ECG) for WCT were prospectively obtained from 120 patients during electrophysiological study. Six physicians with different expertise analyzed the electrocardiographic recordings, and made the diagnosis based on the D12V16 algorithm, that involves the analysis of the predominant polarity of QRS in leads I, II, V1 and V6. The diagnosis was compared with that made using the traditional Brugada algorithm and the “gold-standard” electrophysiological study. Statistical analyses were performed with a significance level of 5% (p<0.05).

**Results:**

According to the EPS study, 82 ECG recordings were VT and 38 SVT-A. Structural heart diseases were present in 71 (86.6%) patients with VT and in 8 (21.1%) with SVT-A. The Brugada algorithm had higher global sensitivity (87.2%), and the D12V16 algorithm had higher global specificity (85.1%) for VT. Both D12V16 and Brugada’s algorithms presented a high positive predictive value (90.9% vs 85.8%, respectively) and similar accuracy (73.8% vs 81.4%, respectively) for the diagnosis of VT. Experienced evaluators were more accurate using Brugada algorithm than the D12V16 algorithm, but the accuracy of both algorithms was similar according to less experienced examiners.

**Conclusion:**

The simplified algorithm may be a useful method to recognize VT in the ECG, especially for less experienced doctors. (Arq Bras Cardiol. 2021; [online].ahead print, PP.0-0)

## Introduction

The recognition of ventricular tachycardia (VT) as the mechanism of wide complex tachycardia (WCT) is an important issue, since an incorrect electrocardiographic (ECG) analysis may lead to inappropriate therapy. In addition, the VT diagnosis also allows more appropriate acute and long-term management of the patient and prevents hospitalizations and unnecessary exams.^1 , 2^

Since the 1960s, many ECG criteria have been proposed in an attempt to differentiate VT from supraventricular tachycardia with aberrant conduction (SVT-A).^3 - 10^Many of them consider that specific measurements in milliseconds result in difficult memorization, low reproducibility of accuracy, and poor clinical applicability. Additionally, in emergency medicine, there is a high rate of discordance between observers, with a low diagnostic accuracy,^11 - 13^that could lead to a harmful treatment.^14^

Therefore, the development of a simple method, preferably visual and easily memorized by resident physicians in the emergency department, regardless of expertise in arrhythmia, could improve clinical decisions.

The discriminative power of ECG leads I, II, V1 and V6 to identify the mechanisms of WCT was initially proposed by Nagi et al.,^15^but it has not been prospectively validated. The aim of this study was to evaluate the diagnostic accuracy of a simplified, easily memorized method for the differential diagnosis of WCT.

### Material and Methods

The research protocol was approved by the Scientific Committee of the Heart Institute, Instituto do Coração - HC-FMUSP, as well as by the Research Ethics Committee of the Messejana - Dr. Carlos Alberto Studart Gomes (HM) – Hospital, under protocol number 336.107.

### Patients and Electrocardiogram

We selected ECG recordings from 120 consecutive patients (one per patient) presented with WCT in the electrophysiological study (EPS) performed in our center between January 2007 and December 2013.

After receiving information about the study, patients willing to participate signed an informed consent form. The study was conducted according to the ethical principles in research involving humans.

WCT was defined by EPS, as a heart rate ≥ 100 bpm in the absence of visible conducted sinus P wave and a QRS duration ≥ 120ms. These ECG tracings were recorded in the EPS and obtained prospectively and consecutively. Patients with WCTs who were taking antiarrhythmic medications, patients with pre-excited tachycardia, and those patients with artificial cardiac pacemakers were excluded. After selecting the 12-lead ECG, we collected the following clinical variables from each patient: age, gender, and presence or absence of structural heart disease.

### The D12V16 algorithm based on the analysis of leads I, II, V1 and V6

All ECG recordings were analyzed according to the simplified algorithm using three steps ( Figure 1 ): (1) In the first step, VT was considered if the four leads (I, II, V1, and V6) had a predominantly negative polarity (R/S ratio < 1). In the absence of these findings, we proceeded to the next step. (2) In the second step, VT was diagnosed if at least three of the four leads displayed a predominantly negative polarity. If this step was not fully completed, we proceeded to the next step. (3) In the third step, VT was diagnosed if at least two of the four leads displayed a predominantly negative polarity (I or V6 necessarily included). If all these steps were not fully completed, the diagnosis assumed was SVT-A. Figures 2 , 3 and 4 present all the steps of the algorithm.

**Figure 1:** – The simplified I, II, V1 and V6 algorithm. Analysis of 120 electrocardiograms for wide QRS tachycardia performed by six observers (total of 720 analyses); each observer analyzed 82 ventricular tachycardia (VT) and 38 supraventricular tachycardia with aberrant conduction (SVT-A); sensitivity, specificity and the number of ECGs that fulfilled the diagnosis are described in the figure.

**Figure 2:** – Example of all four leads with predominantly negative polarity (step 1); these findings have 100% specificity for diagnosis of ventricular tachycardia.

**Figure 3:** – Wide complex tachycardia, using the simplified algorithm; the predominantly negative polarity of three of the four leads suggests ventricular tachycardia.

**Figura 4:** – Example of two predominantly negative polarities (step 3) in four leads (DI, DII, V1 and V6), with inclusion of DI and/or V6 (in this case, both negative).

### ECG Examiners and ECG Analysis

The ECGs were analyzed by three pairs of examiners: (1) two cardiologists with expertise in clinical arrhythmia; (2) two general cardiologists; and (3) two resident physicians of the emergency department. The use of magnifying glasses was allowed if necessary.

The electrocardiograms were analyzed by the six examiners in four different moments: (A) using the Brugada algorithm;^6^(B) using the D12V16 algorithm; (C) repeating the analysis using the Brugada algorithm with clinical information; and (D) repeating the analysis using the D12V16 algorithm with clinical information.

All ECGs recordings were scrambled, and all examiners were blinded to the EPS diagnosis. Clinical information of each electrocardiogram was revealed for the examiners after completing moments A and B.

### Statistical Analysis

The patients’ characteristics are described according to the EPS diagnosis using absolute and relative frequencies. The association between each characteristic and diagnosis was confirmed using chi-square tests. Age of patients was described by mean values and standard deviation and compared according to the diagnosis using unpaired Student’s t-test.

The diagnoses were described according to the four steps of ECG analysis, and the results obtained were compared with the EPS. We calculated diagnostic parameters, e.g. sensitivity (Sn), specificity (Sp), positive predictive value (PPV), negative predictive value (NPV) and accuracy. The agreement of each step/method with the EPS was evaluated by the kappa coefficient. We calculated the corresponding 95% confidence intervals for all of the estimated measures.

The analyses were repeated only for the final diagnoses and the groups were separated according to the examiner’s experience. For statistical analysis of Sn and Sp we used the total amount (n=120) of electrocardiograms interpreted by all the six examiners. Agreements and disagreements with the EPS, considered the gold standard method, were established for each procedure, while the marginal association between the methods was assessed using McNemar’s test. Agreements and disagreements between evaluators were also determined in both methods and these procedures were compared using McNemar’s test. We compared both methods between the groups of evaluators (variability of the traditional method between different evaluators). The Kolmogorov-Smirnov test confirmed the normality of age distribution. The significance level was established at 5% (p<0.05). The software employed in the statistical analysis was the IBM-SPSS for Windows version 20.0.

## Results

### Patient characteristics

Patient characteristics are presented in Table 1 . According to the EPS, 82 patients presented with VT (68%) and 38 had SVT-A; 81 were men, 69.5% of them had VT and 63.2% had SVT-A. Mean age of the patients was 49.1±17.5 years old. Patients with VT were older than SVT-A patients (52.7±16.3 vs. 41.4±17.8 years old; *p* = 0.001). Structural heart disease was detected in 79/120 (65.8%) patients; 71/82 (86.6%) patients with VT, and in 8/38 (21.1%) patients with SVT-A. Patient characteristics are presented in Table 1 .

**Table 1:** – Clinical characteristics of patients diagnosed with supraventricular tachycardia with aberrant (SVT-A) conduction and ventricular tachycardia (VT) according to the electrophysiological study

Variable	SVT-A (n=38)	VT (n=82)	Total (n=120)	p
Male, n (%)	24 (63.2)	57 (69.5)	81 (67.5)	0.489
Heart disease, n (%)	8 (21.1)	71 (86.6)	79 (65.8)	<0.001
Age, years	41.4±17.8	52.7±16.3	49.1±17.5	0.001*
Chi-square test; *Student’s t-test				

### Global analysis of the algorithms

Values of Sn, Sp, PPV, NPV and accuracy, according to each algorithm step, are presented in Table 2 . Six examiners performed a total of 2,880 analyses (720 using the simplified algorithm and the Brugada algorithm, regardless of clinical information). The Sn of the Brugada algorithm was higher than that of the simplified algorithm (87.2% and 68.7%, respectively), while the Sp of the D12V16 algorithm was higher than the Brugada algorithm (85.1% and 68.9%, respectively). The PPV for VT was high for both methods (90.9% in the D12V16 algorithm, and 85.8% in the Brugada algorithm). The diagnostic accuracy was 73.8% using the D12V16 algorithm (k = 0.471; 95% confidence interval [CI], 0.41–0.53), and 81.4% using the Brugada algorithm (k = 0.566; 95% CI, 0.5–0.63). Although not significant, an increase in Sp was observed in the presence of clinical information (90.8% for the simplified algorithm, 73.7% for the Brugada algorithm). A slight increase in accuracy was detected when the Brugada algorithm was applied in the presence of clinical information (k = 0.607; 95%CI, 0.54–0.67). No increase in accuracy was observed using the D12V16 algorithm in the presence or absence of clinical information (k = 0.471; 95% CI, 0.41–0.53; and k = 0.474; 95% CI, 0.42–0.53, respectively).

**Table 2:** – Sensitivity, specificity, positive predictive value (PPV), negative predictive value (NPV) and accuracy of each step of the Brugada algorithm and the D12V16 algorithm with and without clinical characteristics

Criterion	Step	Sensitivity for VT Diagnosis, % (95% CI)	Specificity for VT Diagnosis, % (95% CI)	PPV for VT Diagnosis, % (95% CI)	NPV for VT Diagnosis, % (95% CI)	Accuracy, % (95% CI)
Brugada	Step 1	19.7 (16.3-23.5)	91.7 (87.3-94.9)	83.6 (75.6-89.8)	34.6 (30.8-38.5)	42.5 (38.9-46.1)
	Step 2	48.0 (43.5-52.5)	86.8 (81.8-90.9)	88.7 (84.3-92.3)	43.6 (39.0-48.3)	60.3 (56.7-63.9)
	Step 3	61.8 (57.3-66.1)	83.8 (78.3-88.3)	89.1 (85.4-92.2)	50.4 (45.2-55.5)	68.7 (65.3-72.1)
	Step 4	87.2 (83.9-90.0)	68.9 (62.4-74.8)	85.8 (82.4-88.7)	71.4 (64.9-77.2)	81.4 (78.6-84.2)
D12V16 Algorithm	Step 1	1.0 (0.3-2.4)	100 (98.4-99.0)	100 (47.8-100.0)	31.9 (28.5-42.7)	32.4 (29.0-35.8)
	Step 2	28.0 (24.1-32.2)	97.4 (94.4-99.0)	95.8 (91.2-98.5)	38.5 (34.5-42.7)	50.0 (46.3-53.7)
	Step 3	68.7 (64.4-72.8)	85.1 (79.8-89.4)	90.9 (87.5-93.6)	55.7 (50.4-61.0)	73.8 (70.6-77.0)
Brugada with Clinical Data	Step 1	28.0 (24.1-32.2)	89 (84.2-92.8)	84.7 (78.2-89.8)	36.4 (32.4-40.6)	47.4 (43.8-51.0)
	Step 2	49.4 (44.9-53.9)	87.3 (82.2-91.3)	89.3 (85.0-92.7)	44.4 (39.8-49.2)	61.4 (57.8-65.0)
	Step 3	62.0 (57.5-66.3)	83.8 (78.3-88.3)	89.2 (85.4-92.3)	50.5 (45.4-55.7)	68.9 (65.5-72.3)
	Step 4	87.2 (83.9-90.0)	73.7 (67.5-79.3)	87.7 (84.5-90.5)	72.7 (66.5-78.4)	82.9 (80.1-85.7)
D12V16 Algorithm with Clinical Data	Step 1	1.4 (0.6-2.9)	100 (98.4-100.0)	100 (59.0-100.0)	32.0 (28.6-35.5)	32.7 (29.3-36.1)
	Step 2	27.4 (23.5-31.6)	98.7 (96.2-99.7)	97.8 (93.8-99.5)	38.7 (34.7-42.8)	50.0 (46.3-53.7)
	Step 3	65.0 (60.6-69.3)	90.8 (86.3-94.2)	93.8 (90.7-96.1)	54.6 (49.5-59.7)	73.1 (69.9-76.3)

*values expressed in % (95% confidence interval, 95%CI); VT: ventricular tachycardia.*

### Analysis results by groups of observers

Values of Sn, Sp, PPV, NPV and accuracy by each group of observers are presented in Table 3 . In all three groups, a higher Sn was observed with the Brugada algorithm compared with the D12V16 algorithm, with no difference between the groups. However, Sp was far more evident in the emergency resident physicians (group III) than in the other groups. Higher diagnostic accuracy was observed when cardiology specialized evaluators (groups I and II) used the Brugada algorithm compared to the simplified D12V16 algorithm (84.6% and 85.8% vs. 74.2% and 74.6%, respectively). For emergency medicine resident physicians (group III), the diagnostic accuracy of the two methods was similar (73.7% using the Brugada algorithm, 72.9% using the simplified algorithm). For these evaluators, the D12V16 algorithm showed a higher Sp than the Brugada algorithm (85.5% and 65.8%, respectively). These values were not significantly different in the presence of clinical information ( Table 4 ).

**Table 3:** – Sensitivity, specificity, positive predictive value (PPV), negative predictive value (NPV), and accuracy of each group of observers

Criterion	Group	Sensitivity for VT Diagnosis, % (95% CI)	Specificity for VT Diagnosis, % (95% CI)	PPV for VT Diagnosis, % (95% CI)	NPV for VT Diagnosis, % (95% CI)	Accuracy, % (95% CI)
Brugada	I – Expertise in Arrhythmias	88.4 (82.5-92.9)	76.3 (65.2-85.3)	89.0 (83.1-93.3)	75.3 (64.2-84.4)	84.6 (82.0-87.2)
	II – General Cardiologists	95.7 (91.4-98.3)	64.5 (52.7-75.1)	85.3 (79.4-90.1)	87.5 (75.9-94.8)	85.8 (83.3-88.3)
	III- Emergency Resident Physicians	77.4(70.3-83.6)	65.8 (54.0-76.3)	83.0 (76.1-88.6)	57.5 (46.4-68.0)	73.7 (70.5-76.9)
D12V16 Algorithm	I – Expertise in Arrhythmias	68.9 (61.2-75.9)	85.5 (75.6-92.5)	91.1 (84.7-95.5)	56.0 (46.5-65.2)	74.2 (71.0-77.4)
	II – General Cardiologists	70.1 (62.5-77.0)	84.2 (74.0-91.6)	90.6 (84.1-95.0)	56.6 (47.0-65.9)	74.6 (71.4-77.8)
	III – Emergency Resident Physicians	67.1 (59.3-74.2)	85.5 (75.6-92.5)	90.9 (84.3-95.4)	54.6 (45.2-63.8)	72.9 (69.7-76.1)
Brugada with Clinical Data	I – Expertise in Arrhythmias	86 (79.7-90.9)	81.6 (71,0-89.5)	91.0 (85.3-95.0)	72.9 (62.2-82.0)	84.6 (82.0-87.2)
	II – General Cardiologists	98.2 (94.7-99.6)	72.4 (60.9-82.0)	88.5 (82.9-92.7)	94.8 (85.6-98.9)	90.0 (87.8-92.2)
	III – Emergency Resident Physicians	77.4 (70.3-83.6)	67.1 (55.4-77.5)	83.6 (76.7-89.1)	58.0 (47.0-68.4)	74.1 (70.9-77.3)
D12V16 Algorithm with Clinical Data	I – Expertise in Arrhythmias	65.2 (57.4-72.5)	94.7 (87.1-98.5)	96.4 (91.0-99.0)	55.8 (46.8-64.5)	74.6 (71.4-77.8)
	II – General Cardiologists	70.1 (62.5-77.0)	88.2 (78.7-94.4)	92.7 (86.7-96.6)	57.8 (48.2-66.9)	75.8 (72.7-78.9)
	III – Emergency Resident Physicians	59.8 (51.8-67.3)	89.5 (80.3-95.3)	92.5 (85.7-96.7)	50.7 (42.0-59.5)	69.1 (65.7-72.5)

*Values expressed in % (95% confidence interval, 95%CI).*

**Table 4:** – Agreement and disagreement between the diagnoses made by the Brugada algorithm and the D12V16 algorithm

Evaluation	Brugada	D12V16 Algorithm	p
**Without clinical information**			<0.001
SVT-A agreement, n (%)	157 (21.8)	194 (26.9)	
VT agreement, n (%)	429 (59.6)	338 (46.9)	
Disagreement, n (%)	134 (18.6)	188 (26.2)	
**With clinical information**			<0.001
SVT-A agreement, n (%)	168 (23.3)	207 (28.7)	
VT agreement, n (%)	429 (59.6)	320 (44.4)	
Disagreement, n (%)	123 (17.1)	193 (26.9)	

*Results obtained using McNemar’s test; SVT-A supraventricular tachycardia with aberrant conduction; VT: ventricular tachycardia.*

### Diagnosis agreement between evaluators and methods

Data on diagnostic agreement between the six observers based on the analysis of 120 ECGs are presented in Table 5 . The percentage of disagreement using the Brugada algorithm and the D12V16 algorithm was 60.8% and 30%, respectively. In the presence of clinical information, these values were 51.7% and 30.8%, respectively. This difference was statistically significant (p < 0.001), both with and without clinical information. Figure 5 shows disagreement between algorithms.

**Table 5:** – Agreement and disagreement in the diagnoses made by six evaluators who analyzed 120 electrocardiograms using the Brugada algorithm and the D12V16 algorithm

Evaluation	Brugada	D12V16 Algorithm	p
**Without clinical information**			<0.001
SVT-A agreement, n (%)	8 (6.7)	40 (33.3)	
VT agreement, n (%)	39 (32.5)	44 (36.7)	
Disagreement, n (%)	73 (60.8)	36 (30.0)	
**With clinical information**			<0.001
SVT-A agreement, n (%)	18 (15.0)	43 (35.8)	
VT agreement, n (%)	40 (33.3)	40 (33.4)	
Disagreement, n (%)	62 (51.7)	37 (30.8)	

*Results obtained using McNemar’s test; SVT-A supraventricular tachycardia with aberrant conduction; VT: ventricular tachycardia.*

**Figure 5:** – Example of wide complex tachycardia with diagnostic disagreement – while the simplified algorithm suggests supraventricular tachycardia with aberrant conduction (SVT-A) (step 3), the Brugada algorithm suggests ventricular tachycardia (step 4).

## Discussion

Many of the criteria used for the differential diagnosis of a wide QRS tachycardia are based on peculiar aspects of the electrocardiogram, which are quite difficult to memorize, compromising the clinical applicability and consequently leading to a lack of reproducibility.^7 , 11^When available, consulting with experts is recommended to reduce diagnostic errors and adverse consequences.^2^However, one cannot rely on the presence of “experts” in all emergency department facilities.

In 1999, Nagi et al.^15^proposed values of bipolar (I-II) and precordial (V1-V6) leads for the differential diagnosis of WCT, guided by computer software for ECG analysis. The predominance of negative polarity of at least two of the four leads, with lead I or V6 included, made the diagnosis of TV in 89.2% of cases. Although this is an easily applied technique by non-specialists, it has not been evaluated systematically. In our study, we compared the diagnosis obtained by a DI, DII, V1 and V6 polarity analysis (D12V16 algorithm) with the most frequently used algorithm for WCT diagnosis (Brugada Algorithm), in a significant number of patients. We also evaluated the role of clinical information to improve the correct diagnosis rate, which was applied by the physicians, regardless of their arrhythmia expertise. The Sp of the D12V16 method was higher for VT diagnosis and there were lower disagreement coefficients (greater reproducibility) among observers, with similar accuracy compared to the Brugada algorithm. It is of note that the inclusion of steps 1 and 2, differently from the study by Nagi et al.,^15^increased the Sp of the simplified algorithm (100% and 97%, respectively), thus providing results that are comparable to the presence of atrioventricular dissociation^16 , 17^and steps 1 and 2 of the Brugada algorithm as reported in the original work.^6^

Not surprisingly, it was not possible to reproduce the high accuracy, Sn, and Sp described by Brugada et al.^6^as previously reported by other authors.^4 , 7 , 11 , 18^One reason may rely on the fact that the evaluators who developed the Brugada algorithm were far more experienced than those in other studies. These findings were confirmed by our study, in which the diagnostic accuracy obtained by cardiologists using the Brugada algorithm was higher than that obtained by physicians who were less experienced in arrythmia or cardiology (84.6%, 85.8% and 73.7% by groups I, II, and III, respectively). In group III, the two criteria presented similar results in terms of diagnostic accuracy (73.7% using the Brugada algorithm and 72.9% using the simplified algorithm). In addition, the ECG recordings in our study may also differ from those presented in the paper by Brugada et al.,^6^where no information was obtained about patients showing pre-existing bundle branch block, idiopathic VT, pre-excited SVT, or SVT-A, situations that were not investigated in the present study.^19 , 20^

Although a history of structural heart disease (prior myocardial infarction, angina pectoris, or congestive heart failure) has given a high PPV for VT,^21^no significant increase in accuracy was obtained when the presence of clinical information was added to the analysis using both ECG methods. When comparing the Brugada algorithm according to the presence or absence of clinical information data (k = 0.566; 95% CI, 0.5–0.63 and k = 0.607, 95% CI, 0.54–0.67, respectively), we observed a slight increase in the diagnosis. One possible explanation is that when clinical information was added to “borderline” ECG recordings by the Brugada analysis, the examiners had a reinforcement for the final diagnosis. This fact was not observed for the simplified algorithm, possibly because the analysis of the proposed interpretation of ECG parameters had a “visual” appeal, not demanding extensive analysis.

Ventricular tachycardia is predominant among patients with WCT.^8 , 22^In our study, the prevalence of VT was 68%, while structural heart disease was detected in 87% of patients, confirming the findings in other studies.^5 , 21 , 23^Although the D12V16 and the Brugada algorithms presented similar accuracy for VT diagnosis (rates of 73.8% and 81.4%, respectively), they diverged in terms of Sn and Sp. Therefore, different diagnostic criteria for VT or SVT-A have different diagnostic values. For instance, an evaluator is unlikely to misdiagnose VT using the Brugada algorithm due to its high Sn (87.2%). On the other hand, it is unlikely that VT would be misdiagnosed using the D12V16 algorithm due to its high Sp (85.1%).

The greatest discrepancy was related to Sp of the Brugada method (68.9% in our study vs. 96.5% in the original work published by the authors).^6^Lau et al.,^22^Vereckei et al.,^8^and Griffith et al.^4^also showed lower Sp values using the Brugada algorithm (44%, 73.3%, and 67%, respectively) than the values originally reported.

The greater Sn of the Brugada algorithm has important clinical implications in acute management, since it decreases the possibility that patients with VT are treated as having SVT-A, which would lead to deleterious consequences.^2^On the other hand, the higher PPV (95.8%) of the second step of the D12V16 algorithm could have future implications for clinical practice decision-making and for the development of other discriminatory algorithms to improve diagnostic performance. However, it should be emphasized that when the D12V16 algorithm criteria are not met for VT, it is not possible to conclude that SVT-A is the final diagnosis, due to the relatively low Sn (68.7%) of this new algorithm.

Herbert et al.^11^investigated the variability between observers to differentiate VT from SVT-A using the Brugada algorithm in the emergency medicine scenario. A discrepancy of 22% was observed in the diagnostic prevalence. In our study, among the six evaluators, a higher percentage of disagreement was detected with the use of the Brugada algorithm compared with the D12V16 algorithm (60.8% and 30%, respectively). This difference was statistically significant (p < 0.001) and no reduction was observed in the presence of clinical information (51.7%, and 30.8% respectively; p < 0.001). These results could be explained because D12V16 algorithm has probably a simpler, three-step methodology.

In summary, this study assessed a simple algorithm for the diagnosis of WCT and compared it with the traditional Brugada algorithm.^6^Evaluators with distinct experiences performed the analysis with or without clinical information. Both D12V16 and Brugada algorithms had similar accuracy for the differential diagnosis of wide QRS tachycardia. The Brugada algorithm proved more efficient when applied by cardiologists (groups I and II) as compared with the D12V16 algorithm, a difference that was not observed when it was used by emergency medicine resident physicians (group III). Steps 1 and 2 of the simplified algorithm had a high PPV for VT diagnosis. The Sp and agreement between examiners were higher using the D12V16 algorithm. Additionally, the diagnostic accuracy of the two methods did not increase significantly with the availability of clinical information (presence or absence of heart disease).

### Limitations

One of the main limitations of the study is that it had conducted in a non-emergency environment and the evaluators had no time restrictions for performing the diagnosis, thus eliminating factors such as stress and quick decisions, which are common situations in the emergency room. The observers had all the leads of the ECGs recordings available and not only the specific ones, e.g. bipolar I-II and precordial V1-V6 leads, when using the simplified algorithm or V1–V6 when using the Brugada algorithm. Although this is the method used in the “real world,” this global vision of the electrocardiogram could influence the differential diagnosis. As the final Sn of the algorithm was 68.7% for VT if all three criteria were negative for VT, the misdiagnosis of VT as SVT-A could lead to a deleterious treatment in the emergency setting if only this algorithm is employed.

The number of examiners in each group was small and each one analyzed each electrocardiogram more than one time. We did not analyze the diagnostic variability between observers of the same groups and between different moments of ECG analysis. This could limit the interpretation of the results.

Additionally, other possible causes of WTC were excluded, such as antidromic pre-excited SVT, ventricular paced rhythm, hyperkalemia or other electrolytic disturbances, toxicity for IA and IC antiarrhythmic drugs, and use/abuse of tricyclic antidepressant medications. We also excluded pediatric WCT patients from the analysis. We did not compare the D12V16 algorithm with other algorithms, such as the Pava criterion^24^, both Vereckei algorithms,^8 , 9^the Bayesian approach,^25^and the most recent VT score.^10^External validation of this modified algorithm could bring additional information about the consistence of this algorithm.

## Conclusions

This simple three-step D12V16 algorithm demonstrated high Sp and PPV for VT diagnosis and may represent a simple and useful tool to recognize VT in patients with WCT. The algorithm showed similar accuracy compared with the Brugada algorithm when analyzed by less experienced observers. However, it should be emphasized that when the steps of the D12V16 algorithm are not fulfilled for VT, it is not possible to conclude that SVT-A is the final diagnosis due to the relatively low Sn (68.7%), and thus the risk of “missing” some VT diagnoses. Further studies should be conducted to validate our results.
